# Comparative study of two techniques of laparoscopic burch colposuspension using sutures versus mesh in women with genuine stress urinary incontinence

**DOI:** 10.1080/20905998.2024.2321739

**Published:** 2024-03-07

**Authors:** Basheer N. Elmohamady, Hammouda W. Sherif, Shabieb A. Mohammed, Ahmed H. Mohamed, Abdallah F. Abdelazim

**Affiliations:** Departments of Urology, Benha University, Benha, Egypt

**Keywords:** Laparoscopic Burch Colposuspension, stress urinary incontinence, sutures, mesh, urodynamic analysis, quality of life

## Abstract

**Background:**

To compare the effectiveness and safety of laparoscopic colposuspension using sutures (LCS) versus mesh and staples (LCM) in the treatment of female stress urinary incontinence.

**Methods:**

This randomized study was conducted over a total of 80 women with genuine stress urinary incontinence between January 2020 and April 2022. Women were randomly assigned to the LCS group (*n* = 40) or the LCM group (*n* = 40). They underwent objective evaluations, including a standardized stress test, a 24-hour pad test, and a frequency-volume chart. Subjective assessments were made using a quality-of-life questionnaire.

**Results:**

The LCS group exhibited superior outcomes in PAD test improvement (from 147 [31–304] to 3 [0–300] at 1 year, *p* < 0.001), stress test scores (from 82 [11–153] to 1 [0–124] at 1 year, *p* < 0.001), and mean micturated volume (increase from 294 ± 65 to 321 ± 57 at 1 year, *p* = 0.037) compared to the LCM group. Urodynamic findings revealed a higher Maximum Urethral Closure Pressure in the LCS group (33.1 ± 6.9) versus the LCM group (28.3 ± 6.4, *p* = 0.002). Quality of life improvements were significantly better in the LCS group across various domains. However, the LCM group benefitted from shorter surgery duration, hospital stays, and bladder drainage duration.

**Conclusion:**

LCS demonstrates significant advantages over LCM in treating female stress urinary incontinence, particularly in cure rates and quality of life improvements. Despite the operational benefits of LCM in terms of reduced surgery and recovery times, LCS offers superior therapeutic outcomes.

## Background

Urinary incontinence, a prevalent and often debilitating concern, affects many adult women. Approximately one-third of women of childbearing age experience stress incontinence to some extent [1,2]. This condition arises from the complex interplay of the pelvic floor, bladder, urethra, sphincter, and the neurological system that controls these organs. Damage to any of these components can lead to incontinence [3].

Genuine stress incontinence is characterized by uncontrollable urine loss due to increased intra-abdominal pressure, in the absence of involuntary detrusor contractility, typically caused by urethral sphincteric failure [4]. While various non-surgical treatments address numerous aspects of this disorder, surgical interventions remain pivotal for certain cases [5].

A significant milestone in the treatment of stress urinary incontinence (SUI) was the introduction of the mid-urethral support without tension by Ulmsten and Petros in 1995 [6]. Prior to this, the Burch colposuspension, introduced in 1961, was a major advancement and long considered the gold standard for surgical management of female SUI. Despite its effectiveness and relative safety, it is technically demanding and thus poses challenges in surgical execution [7,8].

Comparative studies between open and laparoscopic colposuspension (LC) have shown varied degrees of success. Traditionally, these procedures have employed sutures [3,9]. However, in 1993, a novel laparoscopic approach using staples and mesh was introduced, aiming to simplify the procedure and reduce the operative time, potentially making it more accessible for surgeons with less laparoscopic experience [10,11]. This introduction sparked debates regarding the effectiveness and safety of using mesh and staples compared to traditional sutures.

Current indications for laparoscopic colposuspension include women with genuine stress incontinence, particularly those who have not responded to or are not suitable for non-surgical treatments [3]. However, the choice between using sutures versus mesh and staples is not straightforward. Each technique has its advantages and potential problems. For instance, suturing, while providing more anatomical support, requires greater surgical skill and may lead to longer operative times [12]. In contrast, the use of mesh and staples, though potentially quicker and easier for the surgeon, raises concerns about long-term complications such as mesh erosion and urinary tract infections [13].

Despite these advancements, there remains a lack of consensus in the literature regarding the optimal surgical approach for SUI, particularly concerning the long-term outcomes and patient quality of life post-surgery. This study, therefore, aims to fill this gap by comparing the effectiveness, safety, and impact on patients’ quality of life between laparoscopic colposuspension with mesh and staples (LCM) and traditional laparoscopic colposuspension using sutures (LCS) in the treatment of female stress urinary incontinence.

## Methods

### Study design and participants

The study comprised 83 genuine stress urinary incontinence (SUI) patients admitted to the Urology Department of Banha University Hospital between January 2020 and April 2022. All patients were subjected to a standardized assessment. Cases with mixed urinary incontinence, recurrent urinary incontinence, cardiac disorders, pulmonary disease, coagulopathy, bleeding tendency, or moderate or severe pelvic organ prolapse, vaginal or uterine pathology, active UTI were excluded.

**Figure 1.**
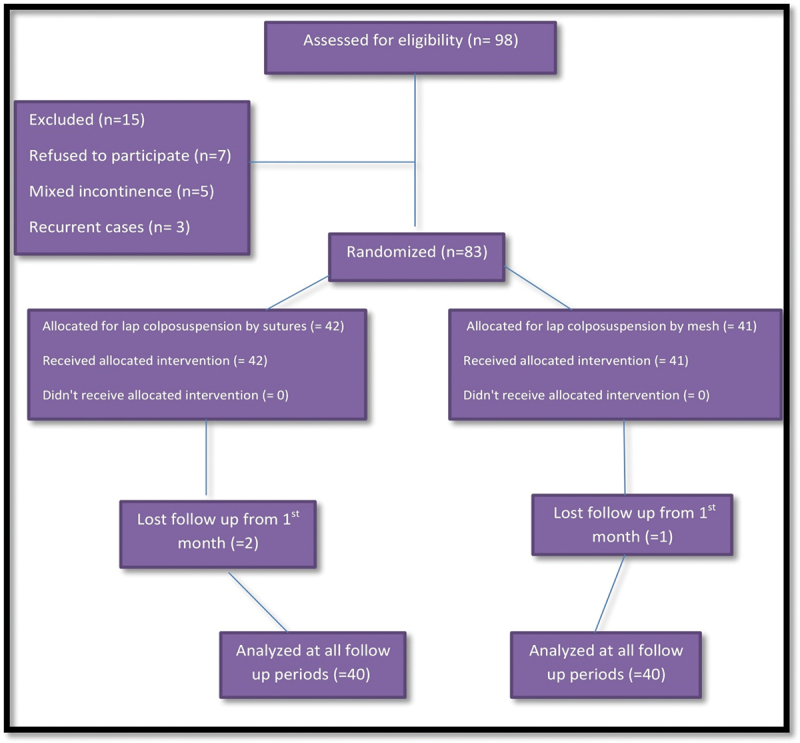
The study flow chart.

Using a computer program, cases were randomly allocated to two groups, 40 patients each (Figure 1). Both groups underwent laparoscopic colposuspension using suture for group A (LCS) and prolene mesh and staples for group B (LCM). Objective assessment was done preoperatively for all patients, including gynecologic examination to exclude vaginal descent, a self-completed 24-h frequency-volume (F/V) chart, a 24-h pad test, and a standardized stress test. During the stress test, the bladder was filled with saline up to half of the cystometric maximal bladder volume. The cough-induced leakage amount was calculated from the additional weight of an incontinence pad used by the patient during the assessment. Urodynamics was done, which included filling cystometry (50 ml/min) to exclude cases with detrusor overactivity and to assess functional bladder capacity, maximal urethral closure pressure (MUCP), and functional urethral length (FUL). Urodynamics are not a must before SUI surgical repair in primary cases, it was done for research purpose.

Subjective assessment was done by a questionnaire (Appendix A) with a series of (yes/no) questions to measure the bother degree from different aspects of incontinence, and a visual analog scale (VAS), which evaluates the effects of incontinence on patients’ quality of life. Two well-experienced surgeons executed all procedures.

### LC technique

LC was done under general anesthesia with the patient in a supine position. After sterilization and draping, a 16 F urethral catheter was inserted, and pneumoperitoneum was established using a verrous needle. Three ports were used: two 5-mm working ports were positioned lateral to each rectus muscle midway between the umbilicus and symphysis pubis, along with one 10-mm umbilical cannula for the laparoscope. Normal saline (300 ml) was introduced into the bladder to identify its upper border. Subsequently, an incision was made in the anterior peritoneum to reach the space of Retzius. The bladder was then emptied, and blunt dissection of the paravaginal fascia was performed, unveiling Cooper’s ligament. The ligament was then cleansed of the fat and the areolar tissue. The assistant’s hand was inserted into the vagina, detecting the Foley balloon and you feel between the thumb and the index fingertips to determine the area needing stapling or stitching to support the bladder neck on either side of the catheter bulb. Two sutures of non reabsorable prolene number 0 were used to repair the paravaginal fascia in group A. Cooper’s ligament and paravaginal tissue were stitched together on the ipsilateral Cooper’s ligament closest to the urethra and two centimeters distal to the bladder neck. The same sutures were performed on the contra-lateral side. In group A, the repair was done by inserting a rectangular piece of prolene mesh about 2 × 4 cm through one of the ports. Using a laparoscopic Kittner dissector or a grasper, the mesh was stapled first to the paravaginal tissues and then to Cooper’s ligament, with the surgeon’s left hand supporting the vagina. The same sutures were performed on the contralateral side. The peritoneum was then reapproximated with the hernia stapler. Cystoscopy was performed to exclude bladder perforation or stapling.

All patients underwent a complete intra-operative assessment to record operative time (from the first skin incision to the last skin suture), blood loss amount, and intraoperative complications such as bladder perforation.

The postoperative assessment was done to record the change in hemoglobin level, hospitalization period, and postoperative complications, such as fever (≥38.5°C), wound infection, urinary tract infection, and urine retention.

The indwelling catheter was removed the next postoperative day, and the residual urine volume was assessed using ultrasonography. Catheter reinsertion was done for two days if the patient developed retention.

All patients were re-assessed at one month, six months, and 1-year postoperatively in the same manner as preoperatively.

### Sample size calculation

The sample size was calculated using G*power software version 3.1.9.2 based on an expected medium to large effect size (d = 0.7) of the six-month mean micturated volume reported in a pilot study done by the authors as a part of the current study. The minimum sample size needed to detect such an effect size is 70 patients (35 per group). The sample size was increased to 80 (40 per group) to compensate for possible loss of follow-up. Alpha and power levels were adjusted at 0.05 and 0.8, respectively.

### Statistical methods

SPSS version 28 (IBM, Armonk, New York, United States) was used for analysis. Shapiro-Wilk test and direct data visualization approaches were employed to assess quantitative data normality. Means with standard deviations or medians with ranges were used to summarize quantitative data, while categorical data were presented as percentages and numbers. The independent t-test or Mann-Whitney U test was used to compare normally and non-normally distributed quantitative data, respectively, between groups. The Chi-square test or Fisher’s exact test, if applicable, was employed for categorical data. Friedman’s test or repeated-measures ANOVA was used for comparing quantitative data within each group. The Bonferroni method was utilized for post hoc analysis to account for multiple comparisons. Each statistical test had two sides. The cutoff for significance was a *p* value of < 0.05.

## Results

No significant differences were observed between both groups in patients’ general characteristics, including age (*p* = 0.678), BMI (*p* = 0.867), parity (*p* = 0.535), and incontinence duration (*p* = 0.328) (Table 1).

**Table 1.** General characteristics of the studied groups.

	Mesh (*n* = 40)	Suture (*n* = 40)	P-value
Age (years)	50 ± 9	51 ± 9	0.678
Body mass index (kg/m^2^)	28.1 ± 2.2	28 ± 2.2	0.867
Parity	3 ± 1	3 ± 1	0.535
Incontinence duration (years)	4 (1–13)	5 (1–11)	0.328

Data were displayed as mean ±SD or median (min-max).

**Figure 2.**
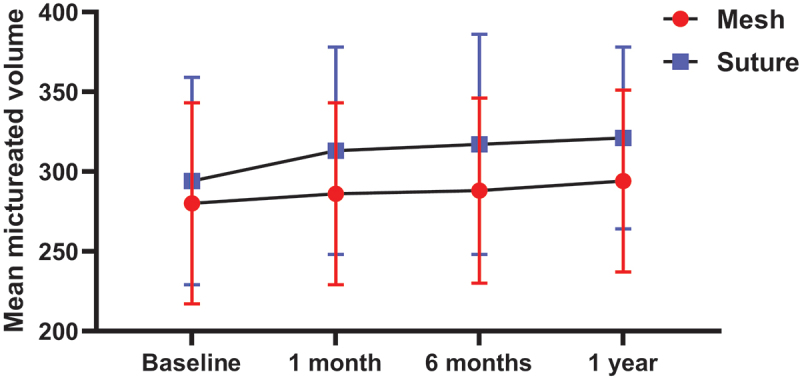
Mean micturated volume at baseline and follow-up in the studied groups.

In patients treated by LCS, significant improvements were detected in the objective assessment parameters, including PAD test, stress test, and mean voided volume compared to patients treated by LCM at 1, 6, and 12 months postoperatively (*p* < 0.001) (Table 2, Figure 2).

**Table 2.** PAD test, stress test and MMV at baseline and follow up in the studied groups.

	Mesh (*n* = 40)	Suture (*n* = 40)	P-value
PAD test			
Baseline	152 (27–302)	147 (31–304)	0.965
At 1 month	6 (0–245) **	2 (0–180) **	<0.001 *
At 6 month	6 (0–260) **	2 (0–220) **	<0.001 *
At 1 year	7 (0–290) **	3 (0–300) **	<0.001 *
P-value	<0.001 *	<0.001*	
Stress test			
Baseline	73 (11–134)	82 (11–153)	0.194
At 1 month	3 (0–105) **	1 (0–95) **	<0.001*
At 6 months	4 (0–119) **	1 (0–102) **	<0.001*
At 1 year	4 (0–128) **	1 (0–124) **	<0.001*
P-value	<0.001*	<0.001*	
Mean micturated volume			
Baseline	280 ± 63	294 ± 65	0.325
At 1 month	286 ± 57	313 ± 65**	0.046*
At 6 months	288 ± 58	317 ± 69**	0.043*
At 1 year	294 ± 57	321 ± 57**	0.037*
P-value	0.108*	0.005*	

Data were displayed as median (min-max); * Significant; ** significantly different from baseline.

The subjective cure rate assessed by VAS, measuring the impact of incontinence on different aspects of QoL, showed a significant decrease in LCS patients than LCM patients at 1, 6, and 12 months postoperatively (Table 3).

**Table 3.** Physical activity, working ability, social life, and sexual life at baseline and follow-up in the studied groups.

	Mesh (*n* = 40)	Suture (*n* = 40)	P-value
Physical activity			
Baseline	75 ± 9	71 ± 10	0.087
One-month	68 ± 12 **	61 ± 12 **	0.013*
Six-months	69 ± 12 **	61 ± 12 **	0.013*
One-year	69 ± 12 **	62 ± 12 **	0.01*
P-value	<0.001 *	<0.001 *	
Working ability			
Baseline	45 ± 10	42 ± 10	0.205
One-month	35 ± 10 **	30 ± 11 **	0.02*
Six-months	36 ± 10 **	30 ± 10 **	0.015*
One-year	37 ± 10 **	31 ± 10 **	0.014*
P-value	<0.001 *	<0.001 *	
Social life			
Baseline	43 ± 10	40 ± 10	0.193
One-month	37 ± 12 **	31 ± 11 **	0.045
Six-months	37 ± 12 **	32 ± 11 **	0.029
One-year	38 ± 11 **	33 ± 11 **	0.027
P-value	<0.001 *	<0.001 *	
Sexual life			
Baseline	29 (6–67)	27 (5–57)	0.522
One-month	25 (3–62) **	20 (1–50) **	0.013
Six-months	20 (2–44) **	12 (1–44) **	<0.001
One-year	19 (2–52) **	13 (1–44) **	0.006
P-value	<0.001 *	<0.001 *	
VAS			
Baseline	75 (50–90)	75 (50–90)	0.942
At 1 month	15 (0–90) **	0 (0–90)**	0.001*
At 6 months	20 (0–90) **	0 (0–90)**	0.003*
At 1 year	20 (0–90) **	0 (0–90)**	0.003*
P-value	<0.001*	<0.001*	

Data were displayed as median (min-max); * Significant; ** significantly different from baseline.

**Figure 3.**
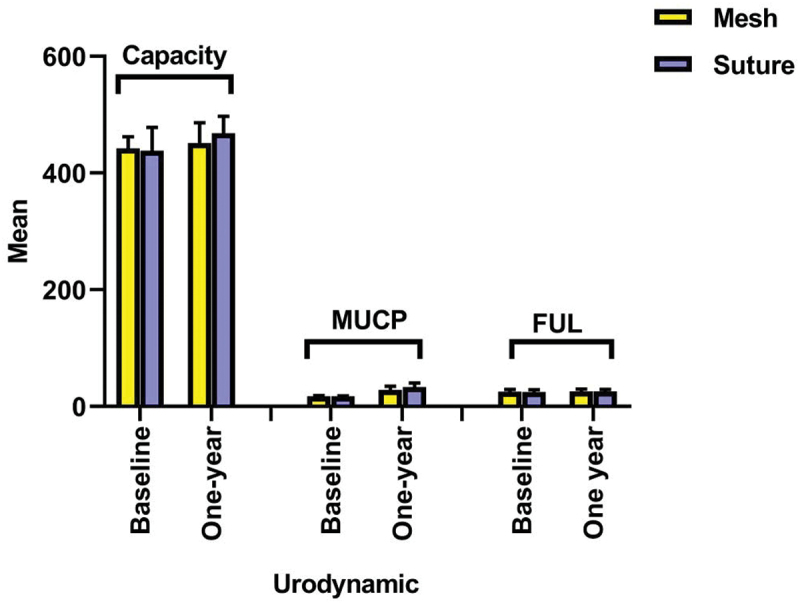
Urodynamic at baseline and 1-year in the studied groups.

Regarding urodynamic findings at one month, the mesh group revealed significantly lower capacity (451 ± 35 vs. 468 ± 29, *p* = 0.023) and MUCP (28.3 ± 6.4 vs. 33.1 ± 6.9, *p* = 0.002). In contrast, no significant difference was reported regarding FUL (*p* = 0.935). Within each group, capacity, MUCP, and FUL significantly increased at one year compared to baseline (Table 4, Figure 3).

**Table 4.** Urodynamic at baseline and 1-year in the studied groups.

	Mesh (*n* = 40)	Suture (*n* = 40)	P-value
Capacity (mL)			
Baseline	442 ± 40	438 ± 40	0.593
One year	451 ± 35	468 ± 29	0.023*
P-value	0.029 *	<0.001*	
MUCP (cm H2O)			
Baseline	17.2 ± 1.3	17 ± 1.3	0.455
One year	28.3 ± 6.4	33.1 ± 6.9	0.002*
P-value	<0.001*	<0.001*	
FUL (cm)			
Baseline	25.2 ± 4.1	24.9 ± 3.8	0.722
One year	25.7 ± 4.1	25.8 ± 3.6	0.935
P-value	<0.001*	<0.001*	

Data were displayed as mean ±SD; * Significant; MUCP: Maximum Urethral Closure Pressure; FUL: functional urethra length.

The mesh group demonstrated significantly less surgery duration (83 ± 17 vs. 93 ± 19 min, *p* = 0.024), hospital stay (median = 2 vs. 3 days, *p* = 0.005), and bladder drainage duration (median = 2 vs. 3, *p* < 0.001). However, no significant variations were reported regarding blood loss (*p* = 0.268) and reduction in hemoglobin (*p* = 0.195).

Complications did not significantly differ between groups, including perforation (*p* = 0.494), retention (*p* = 0.105), urinary tract infection (*p* = 0.06), and wound infection (*p* = 1) (Table 5).

**Table 5.** Operative and postoperative characteristics in the studied groups.

	Mesh (*n* = 40)	Suture (*n* = 40)	P-value
Surgery duration (min)	83 ± 17	93 ± 19	0.024*
Hospital stay	2 (1–4)	3 (1–6)	0.005*
Bladder drainage duration	2 (1–5)	3 (1–8)	<0.001*
Blood loss (ml)	39 (25–119)	43 (22–125)	0.268
Reduction in Hb (mg/dl)	1 ± 0.4	1.1 ± 0.4	0.195
Retention	3 (7.5)	8 (20)	0.105
Urinary tract infection	3 (7.5)	9 (22.5)	0.06
Wound infection	2 (5)	3 (7.5)	1.0
Perforation	0 (0)	2 (5)	0.494

Data were displayed as mean ±SD, n (%), or median (min-max); * Significant.

## Discussion

The evolution of laparoscopic procedures, particularly in the context of stress urinary incontinence (SUI), has been marked by attempts to streamline techniques and reduce the learning curve. The development of devices to replace free-hand suturing, such as the use of polypropylene mesh with tacks or staples, is a testament to this trend [14]. However, these innovations come with their own set of considerations. For instance, while mesh attachment may shorten operating times, it lacks the precision of laparoscopic suturing, which requires significant coordination between endoscopic visualization and manual skill [15].

SUI not only imposes a substantial healthcare burden, but also significantly affects the quality of life (QoL) of those affected [16]. In our study, we observed that patients undergoing laparoscopic colposuspension with sutures (LCS) showed more significant improvements in objective parameters like the PAD test, stress test, and mean micturated volume compared to those treated with mesh and staples (LCM). This aligns with the findings of Ankardal et al. and underscores the potential superiority of LCS in achieving better clinical outcomes [17].

The impact of urinary incontinence on QoL is well-documented, with studies highlighting its influence on social, physical, and emotional well-being [18–21]. Our study adds to this understanding by demonstrating that LCS patients reported greater subjective improvements in QoL across various domains. This finding is corroborated by prior research [17].

Contrasting these benefits, however, is the fact that the LCS procedure is associated with longer surgery times, hospital stays, and bladder drainage durations, as evidenced by our findings and supported by a Cochrane meta-analysis [3]. This trade-off highlights the need for individualized patient care, where the choice of surgical method takes into account both clinical efficacy and operational practicality.

An interesting observation in our study was the increased episodes of urinary retention in the LCS group, more than double that of the LCM group. While not statistically significant, this finding is clinically relevant and warrants further investigation. It may be attributed to the inherent differences in surgical techniques, with suturing potentially leading to more postoperative inflammation and more support to periurethral tissue or bladder outlet obstruction.

Addressing the issue of surgeon experience, it is noteworthy that all procedures in our study were performed by experienced surgeons. This is in contrast to the suggestion that discrepancies in surgical outcomes might be due to variations in surgeon expertise [17]. This highlights the possibility that the differences observed may be more attributable to the intrinsic characteristics of the surgical techniques rather than the skill level of the surgeons.

Our study’s single-center nature limits the generalizability of the findings. Future research, preferably multicenter and with larger sample sizes, is needed to validate our results and explore the long-term efficacy and safety of LCS versus LCM in treating SUI.

Finally, while LCS shows promise in improving clinical outcomes and QoL for patients with SUI, its longer operative times and potential for increased retention episodes present challenges. As surgical techniques evolve, ongoing research is essential to optimize treatment strategies for this prevalent condition.

## Conclusions

Laparoscopic colposuspension, whether utilizing sutures or mesh, has been demonstrated to be practical, safe, and effective in providing positive early functional outcomes. However, significant enhancements in quality of life were observed in the suture group. In contrast, the mesh group benefited from shorter surgery durations, hospital stays, and bladder drainage periods. Comparable outcomes were noted in terms of complications both during and after surgery.

## Supplementary Material

Supplemental Material

## Data Availability

The datasets used and analyzed for the current work are available upon reasonable request from the corresponding author.

## List of abbreviations

FUL: functional urethral length.
F/V: frequency-volume.
LC: laparoscopic colposuspension.
LCM: laparoscopic colposuspension utilizing mesh and staples.
LCS: laparoscopic colposuspension using sutures
MUCP: maximal urethral closure pressure.
OC: open colposuspension Burch method.
SUI: stress urinary incontinence.
VAS: visual analog scale.
