# Supplementary material for: Comparative study of two techniques of laparoscopic burch colposuspension using sutures versus mesh in women with genuine stress urinary incontinence
Source: Arab J Urol. 2024 Mar 7;22(2):121–8. doi: 10.1080/20905998.2024.2321739 (PMC10929671; doi:10.1080/20905998.2024.2321739)
Supplement: Supplemental Material [file TAJU_A_2321739_SM1651.docx]

Appendix .

The following questions evaluate your condition regarding the urine incontinence, urge sypmtoms and their effect on your life. If you have the symptom refered to in the question please demonstrate your answer, based on your own feelings and experince, by a dot placed on a 100 mm line with one end indicating no problem at all and the other end indicating intolerable bother. IF you don’t have the symptome refered to in the question please mark the appropriate box indicating the absence of that sypmtom.
